# Scorpion Stings: Epidemiological, Clinical, and Forensic Medical Evaluation Using University Hospital Data

**DOI:** 10.1590/0037-8682-0208-2025

**Published:** 2025-12-15

**Authors:** Huseyin Kafadar, Neslihan Erin, Ugur Demir, Hasan Buyukaslan

**Affiliations:** 1 Department of Forensic Medicine, Harran University Hospital, Sanliurfa, Turkey . Department of Forensic Medicine Harran University Hospital Sanliurfa Turkey; 2 Department of Emergency Medicine, Harran University Hospital, Sanliurfa, Turkey. Department of Emergency Medicine Harran University Hospital Sanliurfa Turkey

**Keywords:** Scorpions, Envenomation, Scorpion stings

## Abstract

**Background:**

Scorpion stings are a major public health concern in Turkey. This study aims to examine the demographic characteristics, clinical features, and treatment processes of scorpion sting cases in Şanlıurfa Province, located in the Southeastern Anatolia region of Turkey.

**Methods:**

This retrospective study analyzed patient data, including demographic profiles, clinical presentations, and treatment details of scorpion envenomation cases over a 5-year period (June 2019-June 2024), obtained from the Şanlıurfa Harran University Hospital Information Management System.

**Results:**

A total of 1,041 scorpion envenomation cases were analyzed. The mean age was 25.61 ± 18.50 years (mean ± standard deviation), and women (61.47%) comprised the majority of the patients. The age group of 1-17 years (39.38%) was the most affected. Most envenomation cases (63.4%) occurred during summer, and the extremities (29.5%) were the most commonly affected body regions. Most patients (94.1%) received outpatient treatment and were discharged within 24 h. Antivenom was administered to 12.0% of patients, and 15% were subjected to forensic evaluation. One patient (0.1%) developed cardiac arrest during follow-up and died.

**Conclusion:**

This study investigated the clinical and demographic characteristics of scorpion envenomation to highlight the importance of raising public awareness and implementing preventive measures in high-risk regions. It also emphasizes the critical medical and legal importance of examining the forensic aspects of scorpion stings and ensuring that physicians conduct appropriate evaluation in such cases.

## INTRODUCTION

Turkey hosts a significant number of living species because of its diverse climate and geographical location. Among these species, scorpions are particularly common in hot and arid regions and pose significant health risk to humans^1^. Although scorpions are a natural part of the ecosystem, human contact with scorpions can cause serious health problems. The risk of mortality is particularly high in children and patients who lack access to timely treatment^2^.

Scorpions, which belong to the class *Arachnida* within the phylum *Arthropoda*, are covered with a thick chitin layer, and the size of adult scorpions can vary between 11.5 and 220 mm^1^. Of approximately 2,500 scorpion species identified to date, 52 are medically significant to humans^1^^,^^3^. The World Health Organization classifies cases resulting from scorpion stings as *scorpionism* and *scorpion envenomation* and emphasizes these as important yet neglected public health problem in developing countries^1^. These cases, which are common in tropical and subtropical regions such as Turkey, exhibit a wide clinical spectrum ranging from local symptoms to mortality^3^^-^^7^.

Each year, approximately 1.2 million scorpion sting cases are reported worldwide, with more than 3,250 resulting in fatalities^7^^-^^10^. However, the true scale of these figures is not fully reflected due to underreporting^11^. Unlike cases that remain clinically limited to local symptoms, children aged < 10 years and the elderly constitute the highest risk group for systemic toxicity and mortality^12^^,^^13^. The mortality rates among children due to scorpion stings ranges from 5.2% to 8.3%^14^. 

According to the Ministry of Health 2021 report from the National Poison Consultation Center, Turkey reported 3,254 cases of animal exposure in 2018, with 44.87% involving scorpion stings^15^. The two most venomous scorpion species in Turkey, *Leiurus abdullahbayrami* and *Androctonus crassicauda*, are particularly common in the Southeastern Anatolia region^12^. *A. crassicauda*is one of the world's most venomous scorpions. In Şanlıurfa Province, this species is responsible for 50.8% of scorpion stings and constitutes one of the most significant public health threats. Envenomation by both of the species has been reported to cause severe systemic poisoning symptoms^1^.

Scorpion stings should be considered not only as medical emergencies but also as forensic cases. Any incident caused by external factors that leads to deterioration in an individual’s physical or mental health or results in death should be evaluated as a forensic case^16^. The 2019 Turkish Penal Code Guide to Forensic Medical Evaluation of Injury Crimes states that poisoning cases should be examined within the scope of forensic cases^17^. In addition, the Ministry of Health 2005 Circular on Principles to be Followed in the Conduct of Forensic Medical Services emphasizes that physicians must report traumatic lesions in forensic cases^16^^,^^18^^,^^19^. In this context, emergency physicians are obligated to treat patients and conduct forensic evaluations in accordance with the current injury guidelines of the Turkish Penal Code^19^.

Although numerous epidemiological studies on scorpion stings in high-risk regions of Turkey are available^8^, demographic characteristics, anatomical localization, and forensic aspects of cases in Şanlıurfa Province have not been sufficiently examined in the literature.

Therefore, the current study aims to fill this gap in the literature by analyzing demographic data, clinical findings, and forensic medical evaluation processes of scorpion sting cases presented to the emergency department in Şanlıurfa over a 5-year period.

## METHODS

This study retrospectively analyzed hospital records over a 5-year period (June 2019 to June 2024). Scorpion envenomation cases were included in this study. Patient data were obtained from the Şanlıurfa Harran University Hospital Information Management System (HBYS-FONET). These data included age, sex, sting location, forensic evaluation, follow-up duration, need for mechanical ventilation, emergency department admission time, seasonal pattern of sting occurrences, antivenom administration, treatment and follow-up processes, Glasgow Coma Scale (GCS) score, and pregnancy status. Scorpion stings were classified according to their anatomical location, namely head/neck, upper extremities, torso, and lower extremities. Patients were excluded based on the following criteria: those who did not have an International Statistical Classification of Diseases and Related Health Problems-10 code X22, which indicates human contact with scorpions, those who did not receive treatment with scorpion antivenom, or those who had either incomplete or inaccessible medical records in the HBYS. Data were analyzed using IBM SPSS Statistics for Windows (version 21.0; IBM Corp., Armonk, NY, USA). Descriptive statistics are presented as mean and standard deviation (SD) for continuous variables and as case number (n) and percentage (%) for categorical variables. The chi-square test was used for contingency table analyses; when the expected frequency in any cell was less than 5, Fisher’s exact test was used instead. For multi-level contingency tables, the effect size was quantified using the Cramer’s V, and 95% confidence intervals were derived via multinomial bootstrapping with 4,000 resamples. For 2×2 comparisons, we reported the odds ratio (OR), risk ratio (RR), and risk difference (RD) with the corresponding 95% confidence intervals, applying the Haldane-Anscombe correction (0.5 continuity adjustment) when zero cell counts occurred. For continuous variables (e.g., age), multi-group comparisons were conducted using one-way ANOVA; when normality (Shapiro-Wilk test) or homogeneity of variances (Levene’s test) assumptions were not satisfied, the Kruskal-Wallis test was used. All tests were two-sided with an alpha value of 0.05. Primary analyses were conducted using IBM SPSS Statistics (version 21.0). However, advanced contingency table analyses (Cramer's V with bootstrap confidence intervals, OR, RR, and RD calculations) were performed using Python libraries (including SciPy, NumPy, and pandas).

**Ethical Approval:** Harran University Clinical Research Ethics Committee, Date: 22.07.2024, Number: 2024/10.

## RESULTS

This study included 1,041 patients (1,023 scorpion envenomation cases and 18 treated with scorpion antivenom) between June 2019 and June 2024, covering a 5-year period. As shown in Table 1, the cases were categorized according to the clinical department where follow-up and treatment were administered. The table presents a comparison of the patients’ demographic and epidemiological characteristics based on their respective departments. Significant differences were observed between the groups in terms of sex, age, emergency department admission time, sting location, follow-up duration, GCS score, antivenom use, pregnancy status, forensic evaluation, and survival status.

**TABLE 1: Demographic:** and epidemiological characteristics of scorpion sting cases according to follow-up and treatment process.

	All patients (n = 1,041)	Outpatient (n = 881)	Ward (n = 73)	ICU (n = 85)	Referral (n = 2 )	P-value
Sex, n (%)						
Male	401 (38.5)	333 (37.8)	39 (53.4)	28 (32.9)	1 (50)	0.041
Female	640 (61.5)	548 (62.2)	34 (46.6)	57 (67.1)	1 (50)	
Age (years), (mean ± SD) All cases	25.61± 18.50	26.15 ±18.22	8.26 ± 7.40	35.34 ± 18.25	9 ± 8	0.018
Female	27.94 ± 17.97	28.33 ± 18.16	10.41 ± 8.90	35.08 ± 18.79	1 ± 0	
Male	21.9 ± 18.46	22.55 ± 17.75	6.38 ± 5.10	35.85 ± 17.08	17 ± 0	
**ED admission time, n (%)**						
00:00-06:00	117 (11.2)	91 (10.3)	10 (13.7)	16 (18.8)	0 (0)	0.364
06:01-12:00	269 (25.8)	233 (26.4)	17 (23.3)	17 (20.0)	2 (100)	
12:01-18:00	249 (24.0)	216 (24.5)	11 (15.1)	22 (25.9)	0 (0)	
18:01-23:59	406 (39.0)	341 (38.7)	35 (47.9)	30 (35.3)	0 (0)	
**Follow-up duration, n (%)**						
<24 h	982 (94.3)	881 (100)	38 (52.0)	61 (71.8)	2 (100)	<0.001
24-48 h	37 (3.6)	0 (0)	21 (28.8)	16 (18.8)	0 (0)	
48-72 h	10 (1.0)	0 (0)	7 (9.6)	3 (3.5)	0 (0)	
>72 h	12 (1.2)	0 (0)	7 (9.6)	5 (5.9)	0 (0)	
**Envenomation season, n (%)**						
Spring	149 (14.3)	131 (14.9)	11 (15.1)	7 (8.2)	0 (0)	0.058
Summer	660 (63.4)	562 (63.8)	46 (63.0)	50 (58.8)	2 (100)	
Autumn	219 (21.0)	177 (20.1)	16 (21.9)	26 (30.6)	0 (0)	
Winter	13 (1.2)	11 (1.2)	0 (0)	2 (2.4)	0 (0)	
**Envenomation site, n (%)**						
Head/neck	20 (1.9)	14 (1.6)	4 (5.5)	2 (2.35)	0 (0)	<0.001
Upper extremity	170 (16.3)	130 (14.8)	17 (23.3)	23 (27.1)	0 (0)	
Torso	24 (2.3)	22 (2.5)	2 (2.7)	0 (0)	0 (0)	
Lower extremity	137 (13.2)	100 (11.3)	18 (24.7)	19 (22.35)	0 (0)	
Unspecified	690 (66.3)	615 (69.8)	32 (43.8)	41 (48.2)	2 (100)	
**GCS, n (%)**						
3-8	7 (0.7)	0 (0)	3 (4.1)	3 (3.5)	1 (50)	<0.001
9-12	2 (0.2)	0 (0)	1 (1.4)	1 (1.2)	0 (0)	
13-15	1032 (99.1)	881 (100)	69 (94.5)	81 (95.3)	1 (50)	
Antivenom treatment, n (%)	125 (12.0)	74 (8.4)	28 (38.4)	22 (25.9)	1 (50)	<0.001
Forensic evaluation, n (%)	165 (15.9)	124 (14.1)	32 (43.8)	8 (4.7)	1 (50)	<0.001
Mechanical ventilation, n (%)	6 (0.6)	0 (0)	1 (1.36)	4 (4.7)	1 (50)	<0.001
Mortality, n (%)	1(0.1)	0 (0)	0 (0)	1 (1.17)	0 (0)	0.010
Pregnancy (females), n (%)	54 (8.4)	42 (7.7)	1 (2.9)	11 (19.3)	0 (0)	0.005

**ED:** emergency department, **GCS:** Glasgow Coma Scale, **ICU:** intensive care unit, **SD:** standard deviation, **h:** hour.

Among the 1,041 patients, 401 (38.52%) were male, and 640 (61.47%) were female. Females were more frequently affected by envenomation than males (female-to-male ratio: 1.59/1). Among female patients, 54 (8.4%) were pregnant. Patients’ age ranged from 1 to 95 years, with the mean age of 25.61 ± 18.50 years (21.9 ± 18.46 years for males and 27.94 ± 17.97 years for females). The most affected age group was children under 18 years of age (n = 410, 39.38%), whereas 41 cases (3.93%) were individuals aged above 65 years.

Table 2 presents a comparison of the demographic and epidemiological characteristics of scorpion stings according to sex. Significant differences were found with respect to age distribution, emergency department admission time, sting season, follow-up duration, forensic evaluation, and antivenom use. There was a significant association between age group and sex (chi-square(3) = 46.82, *p* < 0.001). The effect size was small-to-moderate (Cramer’s V = 0.21, 95% CI 0.16-0.27). In particular, the likelihood of being male was higher in the 0-17 years group compared with older ages (OR = 2.38, 95% CI 1.84-3.08; RR = 1.68, 95% CI 1.44-1.95; RD = 0.205, 95% CI 0.145-0.266). No notable differences were observed between the two groups regarding mechanical ventilation requirement, survival status, sting location, and GCS score (*p* > 0.05).

**TABLE 2: Demographic:** and epidemiological characteristics of scorpion envenomation cases by sex.

	Male (n = 401)	Female (n = 640)	P-value
**Age group (years), n (%)**			
0-17	209 (52.1)	201 (31.4)	<0.001
18-34	114 (28.4)	240 (37.5)	
35-64	63 (15.7)	173 (27.0)	
>65	15 (3.7)	26 (4.1)	
**Envenomation site, n (%)**			
Head/neck	4 (1.0)	16 (2.5)	0.554
Upper extremity	53 (13.2)	117 (18.3)	
Torso	12 (3.0)	12 (1.9)	
Lower extremity	58 (14.5)	79 (12.3)	
Undetermined	274 (68.3)	416 (65)	
**ED admission time, n (%)**			
00:00-06:00	65 (16.2)	52 (8.1)	0.046
06:01-12:00	90 (22.4)	179 (28.0)	
12:01-18:00	96 (23.9)	153 (23.9)	
18:01-23:59	150 (37.4)	256 (40.0)	
**Envenomation season, n (%)**			
Spring	64 (16.0)	85 (13.3)	0.043
Summer	260 (64.8)	400 (62.5)	
Autumn	73 (18.2)	146 (22.8)	
Winter	4 (1.0)	9 (1.4)	
**GCS, n (%)**			
3-8	2 (0.5)	5 (0.8)	0.662
9-12	1 (0.2)	1 (0.1)	
13-15	398 (99.3)	634 (99.1)	
Forensic evaluation, n (%)	75 (18.7)	90 (14.1)	0.046
Mechanical ventilation, n (%)	1 (0.2)	5 (0.8)	0.270
Mortality, n (%)	0 (0)	1 (0.1)	0.429
Antivenom treatment, n (%)	59 (14.7)	66 (10.3)	0.034
**Total number of patients n = 1,041**			

ED: emergency department, GCS: Glasgow Coma Scale. Table 2: chi-square(3) = 46.82, *p* < 0.001; Cramer’s V = 0.21 (95% CI 0.16-0.27). Comparison of the 0-17 vs. other groups: OR = 2.38 (95% CI 1.84-3.08), RR = 1.68 (95% CI 1.44-1.95), RD = 0.205 (95% CI 0.145-0.266).

Table 3 presents the distribution of scorpion sting cases by age group, comparing demographic and epidemiological features. Significant differences were identified between the groups with respect to sting season, GCS score, mechanical ventilation requirement, forensic evaluation, and antivenom administration. Significant association was observed between sting season and age group (chi-square(9) = 18.39, *p* = 0.031), however, the effect size was small (Cramer’s V = 0.08, 95% CI approximately 0.06-0.10). No notable differences were observed in follow-up/treatment duration, sting location, emergency department admission time, and survival status (*p* > 0.05).

**TABLE 3:** Demographic and epidemiological characteristics of scorpion envenomation cases by age group.

	0-17	18-34	35-64	>65	P-value
**Envenomation season, n (%)**					
Spring	69 (16.8)	43 (12.1)	33 (14.0)	4 (9.8)	0.037
Summer	259 (63.2)	236 (66.7)	143 (60.6)	22 (53.6)	
Autumn	78 (19.0)	74 (20.9)	53 (22.4)	14 (34.1)	
Winter	4 (1.0)	1 (0.3)	7 (3.0)	1 (2.4)	
**Follow-up duration, n (%)**					
<24 h	371 (90.5)	343 (96.9)	230 (97.4)	38 (92.7)	0.002
24-48 h	21 (5.1)	9 (2.5)	5 (2.1)	2 (4.9)	
48-72 h	7 (1.7)	2 (0.6)	0 (0)	1 (2.4)	
>72 h	11 (2.7)	0 (0)	1 (0.4)	0 (0)	
**Envenomation site, n (%)**					
Head/neck	10 (2.4)	6 (1.7)	4 (1.7)	0 (0)	
Upper extremity	62 (15.1)	59 (16.7)	42 (17.8)	7 (17.1)	
Torso	12 (2.9)	10 (2.8)	2 (0.8)	0 (0)	0.571
Lower extremity	58 (14.1)	49 (13.8)	23 (9.7)	7 (17.1)	
Undetermined	268 (65.4)	230 (65.0)	165 (69.9)	27 (65.8)	
**ED admission time, n (%)**					
00:00-06:00	43 (10.5)	57 (16.1)	14 (5.9)	3 (7.3)	
06:01-12:00	111 (27.1)	84 (23.7)	64 (27.1)	10 (24.4)	
12:01-18:00	97 (23.6)	75 (21.2)	64 (27.1)	13 (31.7)	0.230
18:01-23:59	159 (38.8)	138 (39.0)	94 (39.8)	15 (36.6)	
**GCS, n (%)**					
3-8	7 (1.7)	0 (0)	0 (0)	0 (0)	
9-12	2 (0.5)	0 (0)	0 (0)	0 (0)	0.004
13-15	401 (97.8)	354 (100)	236 (100)	41 (100)	
**Treatment setting, n (%)**					
Outpatient	330 (80.5)	310 (87.6)	207 (87.7)	34 (82.9)	
Ward	70 (17.1)	2 (0.6)	1 (0.4)	0 (0)	0.690
ICU	8 (1.9)	42 (11.9)	28 (11.9)	7 (17.1)	
Referral	2 (0.5)	0 (0)	0 (0)	0 (0)	
Mortality, n (%)	1 (0.2)	0 (0)	0 (0)	0 (0)	0.674
Mechanical ventilation, n (%)	6 (1.5)	0 (0)	0 (0)	0 (0)	0.026
Forensic evaluation, n (%)	160 (39.0)	4 (1.1)	1 (0.4)	0 (0)	<0.001
Antivenom treatment, n (%)	93 (22.7)	20 (5.6)	11 (4.7)	1 (2.4)	<0.001
Total number of patients, (n = 1041)	410 (39.4)	354 (34.0)	236 (22.7)	41 (3.9)	

**ED:** emergency department, **GCS:** Glasgow Coma Scale, **ICU:** intensive care unit, **h:** hour. Table 3: (Season × Age group): chi-square(9) = 18.39, *p* = 0.031; Cramer’s V = 0.08 (95% CI approximately 0.06-0.1).

Most sting incidents occurred during the summer (May-July, n = 660, 63.4%), followed by autumn (n = 219, 21.0%), spring (n = 149, 14.3%), and winter (n = 13, 1.2%). Owing to the lack of a pediatric cardiology clinic in July 2019, two pediatric patients (0.2%) were referred, one of whom required mechanical ventilation. One patient who was transferred from a secondary healthcare facility died of cardiorespiratory arrest. The patient, was intubated prior to transfer and originating from a rural area, was admitted in critical condition after experiencing cardiac arrest and subsequent intubation at the initial presenting hospital.

Six patients (0.6%) required mechanical ventilation (endotracheal intubation), including one fatal case. The majority of patients (n = 406, 39%) visited the emergency department between 18:01 and 23:59 O’ clock during the day. Forensic reports were filed for 165 (15.85%) patients.

Sting locations were unspecified in most cases (n = 690, 66.3%). Among localized stings, the upper extremities were most frequently affected (n = 170, 16.3%), followed by the lower extremities (n = 137, 13.2%). Head/neck (n = 20, 1.9%) and torso (n = 24, 2.3%) stings were less common.

Scorpion antivenom was administered in 125 patients (12.0%), including seven referred from primary/secondary centers where antivenom treatment had been initiated. Hospitalization was required for 158 patients (15.17%), of which 99 (62.65%) were discharged within 24 h. Overall, 980 patients (94.1%) had a follow-up duration of less than 24 h.

The GCS score on admission was 13-15 in most patients (n = 1,032, 99.13%).

## DISCUSSION

This retrospective study presents a comprehensive analysis of the demographic characteristics, clinical features, and treatment processes of scorpion envenomation cases presented to a university hospital. In this section, the findings are compared with those reported in the literature, highlighting the clinical and public health significance of this study.

Our study revealed that women were more frequently affected by scorpion envenomation (n = 640, 61.5%), particularly those in the 18-34 age group (n = 240, 37.5%). This difference was statistically significant (*p* < 0.001), which is inconsistent with previous studies, which reported a higher incidence among males^3^^,^^20^. The agricultural socioeconomic structure of the region and higher prevalence of scorpions in rural areas may explain the increased exposure of women actively engaged in farming activities^21^.

The small-to-moderate association between age group and sex (Cramer’s V = 0.21) indicated that statistical significance was accompanied by a non-trivial, though limited, effect size. The increased likelihood of males in the 0-17 years group (OR ≈ 2.38) may help explain the epidemiologic pattern and suggests a need for targeted preventive strategies. In contrast, the season-by-age association was statistically significant but small in magnitude (Cramer’s V ≈ 0.08), implying that seasonal pattern is relatively similar across age strata.

The higher frequency of cases in the 0-17 age group (n = 410, 39.4%) can be explained by greater vulnerability of children to environmental hazards. Notably, males predominated this age group (n = 209, 52.1%), which aligns with the literature suggesting that boys spend more time outdoors and are thus more exposed to scorpion habitats.

Similar to previous studies^22^^,^^23^, our study found that both upper and lower extremities are the most common sites of envenomation. Scorpions typically sting when accidentally in contact or when they perceive a threat. Therefore, the extremities are more likely to be affected during daily activities, whereas the head and neck are more vulnerable during sleep or at rest^24^. Frequent use of the extremities in daily activities also explains this pattern. However, we observed significant differences in sting locations based on the treatment and follow-up processes (*p* < 0.001). In intensive care unit (ICU) cases, the upper extremities were most commonly affected, whereas in ward admissions, the lower extremities were predominantly affected, followed by the head/neck and torso.

The higher incidence of stings during the summer months in the 0-17 age group (n = 259, 63.2%) may be related to children spending more time outdoors during this period^12^. Across all age groups, cases most frequently occurred between 18:01 and 23:59 (n = 406, 39.0%) and increased during the summer months (n = 660, 63.4%), which can be explained by scorpions being more active in warm weather and at night^1^^,^^6^^,^^22^^,^^25^. However, 232 cases (22.3%) occurred in autumn and winter, possibly due to increased scorpion activity during the rainy season, particularly when the monthly rainfall exceeded 30 mm^9^.

Studies have indicated that scorpion envenomation typically follows a mild clinical course, enabling early discharge^26^. In our study, most patients across all age groups and both sexes were treated as outpatients (n = 881, 84.6%) and discharged within 24 h. These findings align with previous study results^8^^,^^12^^,^^15^^,^^25^. Even cases requiring hospitalization typically had shorter stays of less than 24 h (**Table 1**), potentially attributed to prompt and appropriate intervention and improvements in critical care services in the healthcare system. The mean age of the study population was 25.61 years (standard deviation ± 18.50), with pediatric patients requiring longer hospitalization. Mechanical ventilation was required in 0.6% (n = 6) of the patients, with notable differences by treatment process and age group.

Studies have reported that scorpion envenomation in children can cause severe complications, such as heart failure and pulmonary edema, increasing demand for ICU care, with symptom severity inversely correlated with age and body weight^27^. In our study, all patients requiring mechanical ventilation were pediatric (0-17 years old). However, ICU admission rate among children was lower than expected. Although previous studies indicate a higher need for ICU care in children^28^, the lower rate in our study may reflect regional variations or different treatment approaches^1^. By contrast, patients aged 65 years and older had the highest ICU admission rate (n = 7, 17.1%). Although antivenom is a crucial treatment for scorpion envenomation, the literature reveals conflicting views regarding its use and effectiveness. Some studies advocate the use of antivenom in severe cases requiring ICU care^23^, whereas others raise doubts about its efficacy in milder cases^9^^,^^20^^,^^29^. For example, a 1995 Brazilian study of 7,000 envenomation cases reported 1% mortality despite antivenom use^29^. 

In our study, two pediatric patients developed cardiac arrest after antivenom administration and required resuscitation, and one of them died. Of all cases in this study, 12.0% received antivenom. This relatively low rate may reflect the small proportion of patients with advanced disease in the study population. However, increased antivenom use in ICU (n = 22, 25.9%) and ward admissions (n = 28, 38.4%, *p* < 0.001) suggests that it is preferred in severe cases. The 0-17 year age group had the highest antivenom administration rate (n = 93, 22.7%), likely due to more severe symptoms in children than in other age groups^27^.

The mortality rate was very low (0.1%) (Table 1), indicating that scorpion envenomation can be managed effectively with proper clinical care. However, the mortality risk was higher in cases requiring ICU care (1.17%, *p* < 0.05) (Table 1), underscoring the importance of critical care support in severe cases.

The high ICU admission rate among pregnant patients (19.3%, *p* < 0.001) suggests that this population requires particularly vigilant management. The literature reports severe envenomation outcomes in pregnant women, including fetal loss, premature delivery, and placental abruption^26^. However, we did not observe such complications in pregnant patients in this study.

Most cases had GCS score of 13-15, which was consistent across age groups (0-17 years: n = 401, 97.8%; 18-34 years: n = 354, 100%; 35-64 years: n = 236, 100%; >65 years: n = 41, 100%; *p* = 0.004), indicating that scorpion envenomation rarely causes loss of consciousness. This finding aligns with literature reporting predominantly normal GCS scores^30^^,^^31^. However, more cases with low GCS score (<12) (Table 3) were observed in the 0-17 age group (n = 9, 2.2%), possibly reflecting greater neurological susceptibility in children^9^. Moreover, ICU cases frequently showed low GCS score (3-8: n = 3, 3.5%, *p* < 0.001).

Although some emergency department cases meet the forensic criteria, reporting is often incomplete or inadequate^32^. In our study, 15.9% of envenomation cases underwent forensic evaluation. Notably, the 0-17 age group had significantly higher forensic evaluation rate (n = 160, 39.0%; *p* < 0.001) than that of the other age groups, suggesting greater legal scrutiny of pediatric cases. Our review found no prior Turkish data on forensic assessments of scorpion envenomation, making this study the first, to the best of our knowledge, to perform such analysis. Unreported cases present ethical and legal risks, and physicians must fulfill their forensic obligations to avoid legal consequences^32^. Under Article 280 of the Turkish Penal Code (2005), failure to report such cases can result in imprisonment^33^. Therefore, clinicians must balance treatment with forensic evaluation responsibilities. 

In conclusion, this study shows that the majority of scorpion envenomation cases follow a mild clinical course and can be treated on an outpatient basis. The higher incidence of envenomation during the summer months and nighttime highlights the importance of raising public awareness to prevent incidents. Although antivenom therapy remains an effective option for severe cases, comprehensive research is required to determine its impact on mortality and complications. Furthermore, physicians need to perform forensic responsibilities and ensure thorough documentation. Owing to the retrospective nature of this study, it relied on available hospital records, which may lack detailed clinical parameters. Furthermore, the lack of autopsy data for fatal cases and the unidentified scorpion species in most cases are other limitations inherent to this study design. Future prospective studies are crucial to comprehensively monitor clinical progression, laboratory findings, and responses to treatments such as antivenom in real time. Additionally, identifying the exact scorpion species involved in stings and correlating them with clinical severity would significantly enhance our understanding and management of envenomation in this region.

## Data Availability

The datasets generated and analyzed during the current study are available from the corresponding author upon reasonable request.

## References

[1] 1. Filazi A, Özkan Ö. The history of scorpion serum in Turkey. Turk Hijyen Den Biyol Derg. 2021;78(1):107-16. doi:10.5505/TurkHijyen.2020.69937

[2] 2. Koyuncu E, Balcı O, Kılıç A, Almaz V, Kaya C, Yılmaz K, et al. Çocuk acil servisine akrep sokması nedeniyle başvuran olguların değerlendirilmesi. Harran Univ Tip Fak Derg. 2015;12(3):348-55.

[3] 3. Rahimi M, Shadnia S, Mirzaei Nasab R, Soltaninejad K. Scorpion stings in Tehran Province, Iran: a seven-year hospital-based study. Int J Med Toxicol Forensic Med. 2021;10(4):30274. doi:10.32598/ijmtfm.v10i4.30274

[4] 4. Fereidooni R, Shirzadi S, Ayatizadeh SH, Bahloul M, Tavangar A, Zomorodian SA, et al. Scorpion envenomation-associated myocarditis: a systematic review. PLoS Negl Trop Dis. 2023;17(4):e0011219. doi:10.1371/journal.pntd.0011219 10.1371/journal.pntd.0011219 PMC10075437 37018229

[5] 5. Feola A, Perrone MA, Piscopo A, Casella F, Della Pietra B, Di Mizio G. Autopsy findings in case of fatal scorpion sting: a systematic review of the literature. Healthcare (Basel). 2020;8(3):325. doi:10.3390/healthcare8030325 10.3390/healthcare8030325 PMC7551928 32899951

[6] 6. Isbister GK, Volschenk ES, Seymour JE. Scorpion stings in Australia: five definite stings and a review. Intern Med J. 2004;34(7):427-30. doi:10.1111/j.1445-5994.2004.00625.x 10.1111/j.1445-5994.2004.00625.x 15271178

[7] 7. Carrera-Fernández MC, Herrera-Martínez M, Ordaz-Hernández A, Arreaga-González HM. Medicinal plants from Mexico used in the treatment of scorpion sting. Toxicon. 2023;230:107172. doi:10.1016/j.toxicon.2023.107172 10.1016/j.toxicon.2023.107172 37211060

[8] 8. Çelik E, Dursun A. Akrep sokması nedeniyle başvuran çocuk olguların değerlendirilmesi: bir üniversite hastanesi deneyimi. Bozok Tip Derg. 2020;10(2):60-6. doi:10.16919/bozoktip.580016

[9] 9. Godoy DA, Badenes R, Seifi S, Salehi S, Seifi A. Neurological and systemic manifestations of severe scorpion envenomation. Cureus. 2021;13(4):e14715. doi:10.7759/cureus.14715 10.7759/cureus.14715 PMC8158070 34055554

[10] 10. Pradeep YKL, Bhogaraju VK, Pathania M, Rathaur VK, Kant R. Uncommon presentation of scorpion sting at teaching hospital. J Family Med Prim Care. 2020;9(5):2562-5. doi:10.4103/jfmpc.jfmpc_310_20 10.4103/jfmpc.jfmpc_310_20 PMC7380751 32754547

[11] 11. Kumar L, Naik SK, Agarwal SS, Bastia BK. Autopsy diagnosis of a death due to scorpion stinging: a case report. J Forensic Leg Med. 2012;19(8):494-6. doi:10.1016/j.jflm.2012.02.028 10.1016/j.jflm.2012.02.028 23084316

[12] 12. Çiçek A, Demir G, Elibol P, Bardak Ş, Gökalp G, Nalbant T, et al. Akrep sokması vakalarında prognozu öngören belirteçler: üçüncü basamak çocuk acil servis kesitsel çalışması. Turk Klin J Pediatr. 2021;30(3):177-83. doi:10.5336/pediatr.2021-82083

[13] 13. Abd El-Aziz FEA, El Shehaby DM, Elghazally SA, Hetta HF. Toxicological and epidemiological studies of scorpion sting cases and morphological characterization of scorpions (*Leiurus quinquestriatus*and*Androctonus crassicauda*) in Luxor, Egypt. Toxicol Rep. 2019;6:329-35. doi:10.1016/j.toxrep.2019.03.004 10.1016/j.toxrep.2019.03.004 PMC6479097 31049294

[14] 14. Canpolat M, Per H, Gümüş H, Narin N, Kumandaş S. Akrep sokması sonucu gelişen nadir bir komplikasyon konvülsiyon. Erciyes Med J. 2008;30(3):175-9.

[15] 15. Türkiye Cumhuriyeti Sağlık Bakanlığı Halk Sağlığı Genel Müdürlüğü, Ulusal Zehir Danışma Merkezi. Ulusal Zehir Danışma Merkezi (UZEM) akrep zehirlenmeleri raporu 2014-2018 yılları. Ankara: T.C. Sağlık Bakanlığı; 2021. p. 573-650.

[16] 16. Türkmen N, Akgöz S, Çoltu A, Ergin N. Uludağ Üniversitesi Tıp Fakültesi acil servisine başvuran adli olguların değerlendirilmesi. Uludag Univ Tip Fak Derg. 2005;31(1):25-9.

[17] 17. Turkish Medico-Legal Association, Council of Forensic Medicine, Forensic Medicine Association. Guide for forensic medical evaluation of injury crimes defined in the Turkish Penal Code. In: Balcı Y, Çolak B, Gürpınar K, Anolay NN, editors. Ankara: Council of Forensic Medicine; 2019 Jun [cited 2025 Apr 07]. Available from: Available from: https://www.atk.gov.tr/tckyaralama24-06-19.pdf

[18] 18. Sarı S, Uyanıkoğlu A. Şanlıurfa yöresi intoksikasyon olguları ve karaciğer toksisitesinin değerlendirilmesi. Harran Univ Tip Fak Derg. 2021;18(3):456-9. doi:10.35440/hutfd.931668

[19] 19. Republic of Turkey Ministry of Health, General Directorate of Public Health. Circular on the principles for forensic medicine services (Circular No: 2005/143) [Internet]. Ankara: Ministry of Health; 2005 Sep 22 [cited 2025 Apr 7]. Available from: Available from: https://hsgm.saglik.gov.tr/tr/mevzuat/genelgeler/115-2005-143-say%C4%B1l%C4%B1-adli-tabiplik-hizmetlerinin-y%C3%BCr%C3%BClmesinde-uyulacak-esaslar-konulu-genelge.html

[20] 20. Al Abri S, Al Rumhi M, Al Mahruqi G, Shakir AS. Scorpion sting management at tertiary and secondary care emergency departments. Oman Med J. 2019;34(1):9-13. doi:10.5001/omj.2019.02 10.5001/omj.2019.02 PMC6330186 30671178

[21] 21. Yiğit B, Kara FÖ. Tarımda kadın emeği: Şanlıurfa ili örneği. Soc Ment Res Think J. 2023;9(77):5034-41. doi:10.29228/smryj.72948

[22] 22. Bosnak M, Ece A, Yolbas I, Bosnak V, Kaplan M, Gurkan F. Scorpion sting envenomation in children in southeast Turkey. Wilderness Environ Med. 2009;20(2):118-24. doi:10.1580/07-WEME-OR-098RR3.1 10.1580/07-WEME-OR-098RR3.1 19594203

[23] 23. Duran E, Binici O, Atlas A, Pehlivan VF, Pehlivan B, Büyükfırat E, et al. Şanlıurfa'da genel yoğun bakım ünitemizde akrep sokmalarına yaklaşım. Harran Univ Med J. 2023;20(1):203-7.

[24] 24. Abourazzak S, Achour S, Arqam L, Atmani S, Chaouki S, Semlali I, et al. Epidemiological and clinical characteristics of scorpion stings in children in Fez, Morocco. J Venom Anim Toxins Incl Trop Dis. 2009;15(2):255-67. doi:10.1590/S1678-91992009000200008

[25] 25. Sanaei-Zadeh H, Marashi SM, Dehghani R. Epidemiological and clinical characteristics of scorpionism in Shiraz (2012-2016); development of a clinical severity grading for Iranian scorpion envenomation. Med J Islam Repub Iran. 2017;31:27. doi:10.18869/mjiri.31.27 10.18869/mjiri.31.27 PMC5804458 29445656

[26] 26. Abroug F, Ouanes-Besbes L, Tilouche N, Elatrous S. Scorpion envenomation: state of the art. Intensive Care Med. 2020;46(3):401-10. doi:10.1007/s00134-020-05924-8 10.1007/s00134-020-05924-8 32125457

[27] 27. Çağlar A, Köse H, Babayiğit A, Öner T, Duman M. Predictive factors for determining the clinical severity of pediatric scorpion envenomation cases in southeastern Turkey. Wilderness Environ Med. 2015;26(4):451-8. doi:10.1016/j.wem.2015.04.005 10.1016/j.wem.2015.04.005 26432426

[28] 28. Zengin N, Anıl M, Anıl AB. Ege Bölgesinde çocuklarda akrep sokmasının klinik özellikleri: bir eğitim ve araştırma hastanesi deneyimi. J Pediatr Emerg Intensive Care Med. 2016;3(2):69-75. doi:10.4274/cayd.52714

[29] 29. Abroug F, ElAtrous S, Nouira S, Haguiga H, Touzi N, Bouchoucha S. Serotherapy in scorpion envenomation: a randomised controlled trial. Lancet. 1999;354(9182):906-9. doi:10.1016/S0140-6736(98)12083-4 10.1016/s0140-6736(98)12083-4 10489950

[30] 30. Hamdaoui A, Turki H, Lassoued T, Samet A, Rejeb I. Managing scorpion envenomations: a Gabes emergency department case study of 60 patients. Tunis Med. 2024;102(9):529-36. doi:10.62438/tunismed.v102i9.4885 10.62438/tunismed.v102i9.4885 PMC11450744 39287344

[31] 31. Raqeeb MK, Fathul H, Ahmad S. A retrospective analysis of scorpion envenomation cases treated at Rozhhalat Emergency Hospital in Erbil City in 2018. J Med Surg Pract. 2021;7(2):153-67.

[32] 32. Hakkyomaz H, Keten HS, Artuç S, Üçer H, Bozkurt S, Okumuş M, et al. Acil serviste düzenlenen adli raporların Türk Ceza Kanunu kapsamında değerlendirilmesi. J Kartal TR. 2014;25(3):177-80. doi:10.5505/jkartaltr.2014.36693

[33] 33. Turkish Penal Code. Law No. 5237. Official Gazette Date: 2004 Oct 12; No: 25611 [cited 2025 Apr 8]. Available from: Available from: https://www.mevzuat.gov.tr/mevzuatmetin/1.5.5237.pdf

